# Melatonin signalling in Schwann cells during neuroregeneration

**DOI:** 10.3389/fcell.2022.999322

**Published:** 2022-10-10

**Authors:** Andrii Klymenko, David Lutz

**Affiliations:** Department of Neuroanatomy and Molecular Brain Research, Ruhr University Bochum, Bochum, Germany

**Keywords:** extracellular matrix reorganisation, basal lamina, melatonin receptors, Schwann cells, circadian rhythm, peripheral nerve injury

## Abstract

It has widely been thought that in the process of nerve regeneration Schwann cells populate the injury site with myelinating, non–myelinating, phagocytic, repair, and mesenchyme–like phenotypes. It is now clear that the Schwann cells modify their shape and basal lamina as to accommodate re–growing axons, at the same time clear myelin debris generated upon injury, and regulate expression of extracellular matrix proteins at and around the lesion site. Such a remarkable plasticity may follow an intrinsic functional rhythm or a systemic circadian clock matching the demands of accurate timing and precision of signalling cascades in the regenerating nervous system. Schwann cells react to changes in the external circadian clock clues and to the Zeitgeber hormone melatonin by altering their plasticity. This raises the question of whether melatonin regulates Schwann cell activity during neurorepair and if circadian control and rhythmicity of Schwann cell functions are vital aspects of neuroregeneration. Here, we have focused on different schools of thought and emerging concepts of melatonin–mediated signalling in Schwann cells underlying peripheral nerve regeneration and discuss circadian rhythmicity as a possible component of neurorepair.

## Introduction

A wide body of research conducted over the last decades has demonstrated that the circadian hormone melatonin (N-acetyl-5-methoxytryptamine) plays a significant role in oligodendrogenesis (Breton et al., 2021), regulation of neural stem cell proliferation (Gengatharan et al., 2021), peripheral nerve regeneration, and re–myelination after injury of the central nervous system (Naseem and Parvez, 2014). Beneficial effects of melatonin on Schwann cell functions reported *in vitro* and during nerve regeneration (Chang et al., 2014; Rateb et al., 2017; Moharrami Kasmaie et al., 2019; Tiong et al., 2020; Gengatharan et al., 2021) imply that the Schwann cells are sensitive to intrinsic/extrinsic clock alterations and render rhythmicity as a possible factor of neurorepair. However, the experimental evidence is scarce. In this review, we have explored the concept of melatonin–mediated signalling in Schwann cells and circadian rhythmicity as a component of nerve regeneration.

**FIGURE 1:**
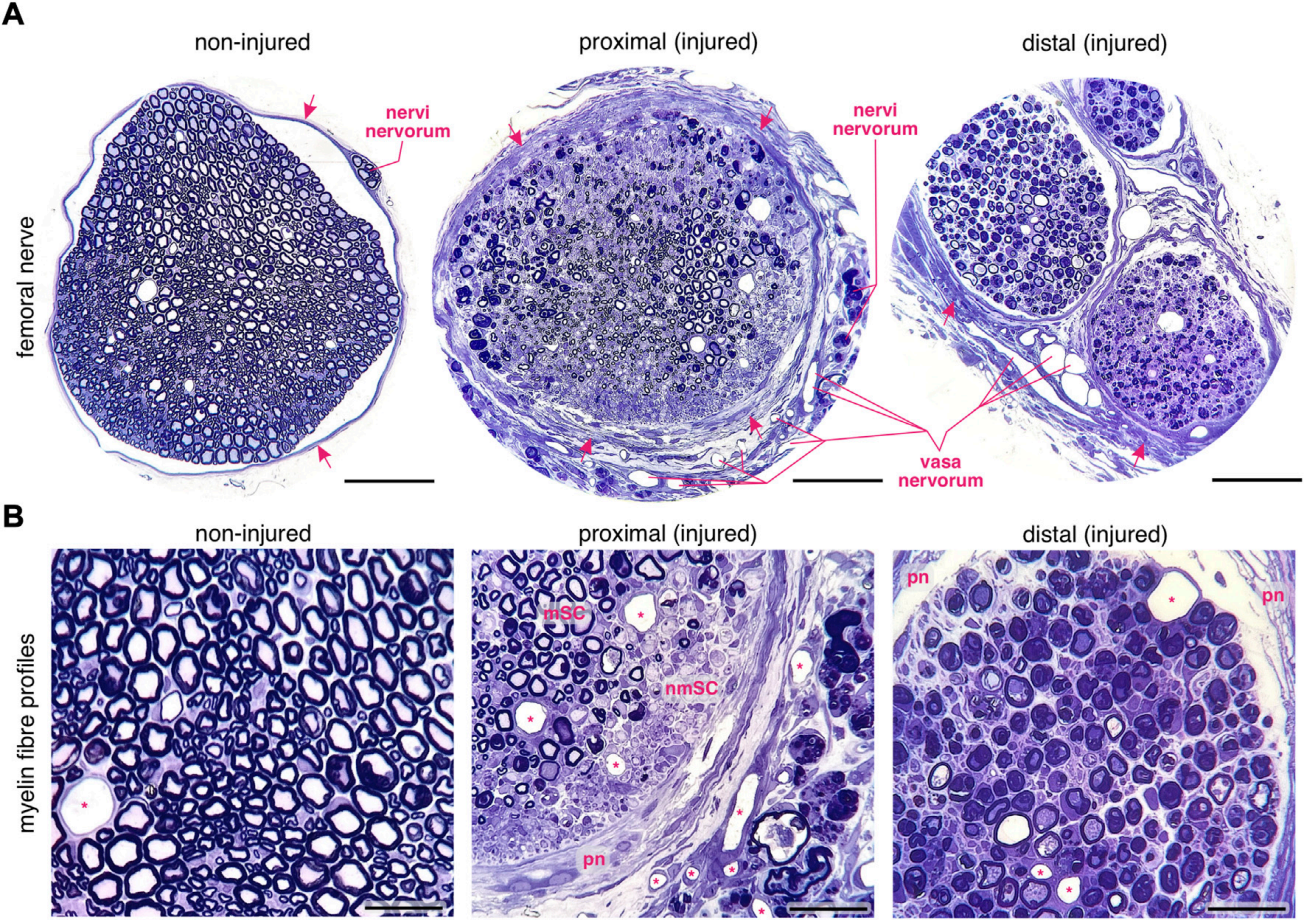
Changes in myelin fibre profiles of the regenerating murine femoral nerve after transection. **(A)** Left: a transversal profile of an intact femoral nerve. Arrows indicate the perineural sheath. Middle: a transversal profile of the proximal stump of the regenerating femoral nerve. Note the thick perineurium (arrows), the enhanced angiogenesis and the degeneration of *nervi nervorum* after transection. Right: the distal stump of the transected femoral nerve. Scale bar, 100 µm. **(B)** Left: the intact femoral nerve is composed of homogeneously distributed myelinated and non–myelinated fibres. Middle: sprouting in myelinated (myelinating Schwann cells, mSC) and non–myelinated (non–myelinating Schwann cells, nmSC) areas in the regenerating proximal nerve stump. The thick perineurium (pn) and newly formed blood vessels (asterisks) are visible. Right: distally to the lesion site, myelin decomposition occurs in variable forms within the Schwann cells. Scale bar, 30 µm. Staining: toluidine blue. Specimen: 75–nm–thin sections of resin–embedded non-injured nerves *versus* injured nerves 10 days after transection in C57Bl6/J mice.

**FIGURE 2:**
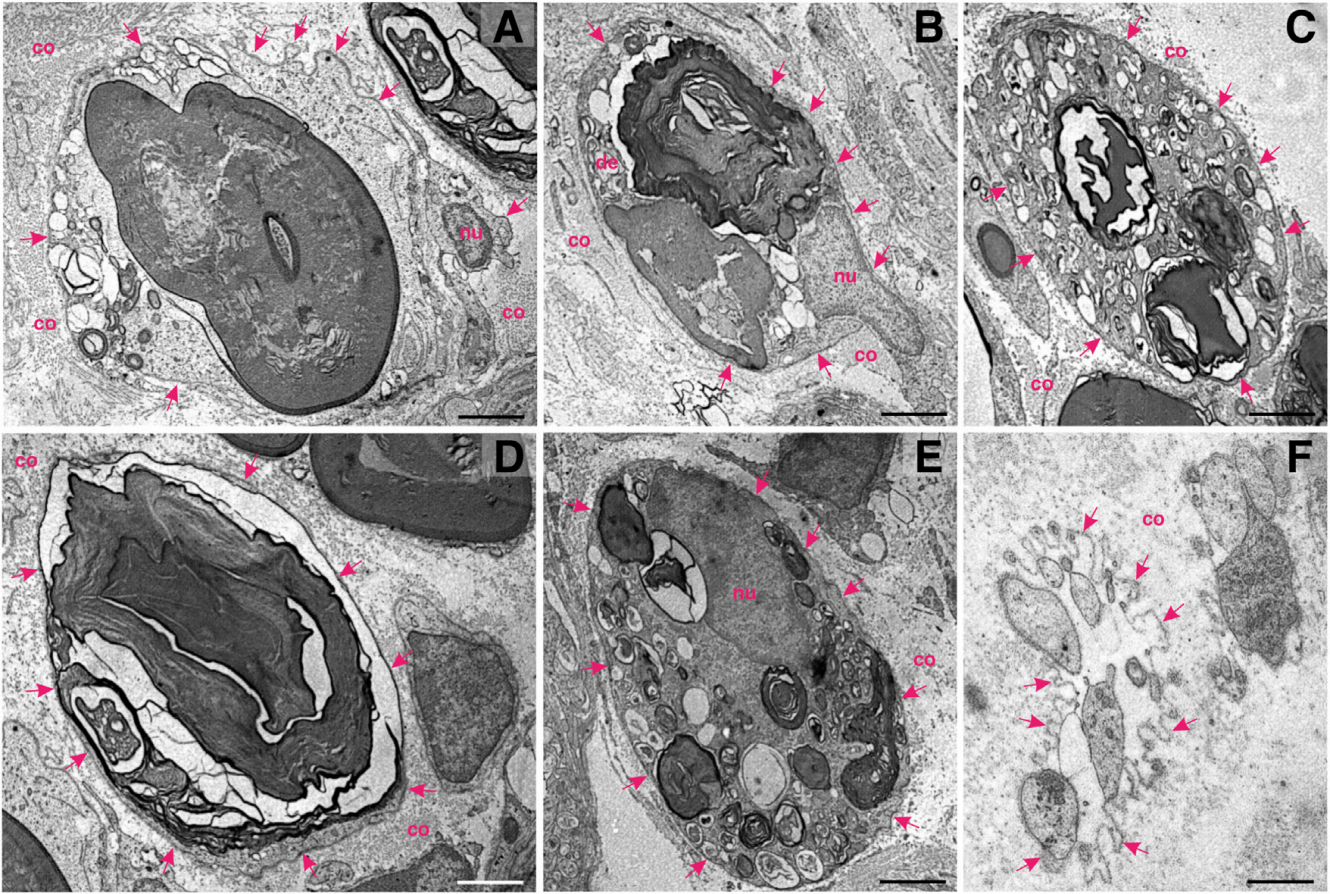
Schwann cells at work—ultrastructural patterns of myelin decomposition in the distal stump of a transected murine femoral nerve. **(A)** A degenerating axon amidst compacted myelin within the Schwann cell cytoplasm. Multiple proteolytic vesicles are visible in the cytoplasmic rim. A basal lamina (arrows) and collagen (co) isolate the Schwann cell from the extracellular matrix. **(B)** Ovoid figures of partially degraded myelin and peripheral vesicular fusion at multiple sites. The nucleus (nu) of the Schwann cell is visible. **(C)** A multitude of cytoplasmic vesicles and a few compartments of degraded myelin. The basal lamina (arrows) is an important border, which isolates the dynamic cell interior from the surrounding. **(D)** Decomposition of a huge mass of myelin within a myelinating Schwann cell. **(E)** Portioning and proteolytic degradation of myelin (concentric myelin lamellae are visible). **(F)** Star–like appearance of the basal lamina (arrows) harbouring the processes of the Schwann cells in the cross–section, also known as “Büngner’s band.” This structure is able to accommodate and guide re–growing axons; nu—nucleus. Staining: osmium tetroxide, potassium (III) hexacyanoferrate and post–contrasting with lead nitrate and uranyl acetate. Specimen: 55–nm–thin sections of resin–embedded distal nerve stumps, 10 days after transection performed in C57Bl6/J mice. Scale bar, 2 µm.

**FIGURE 3:**
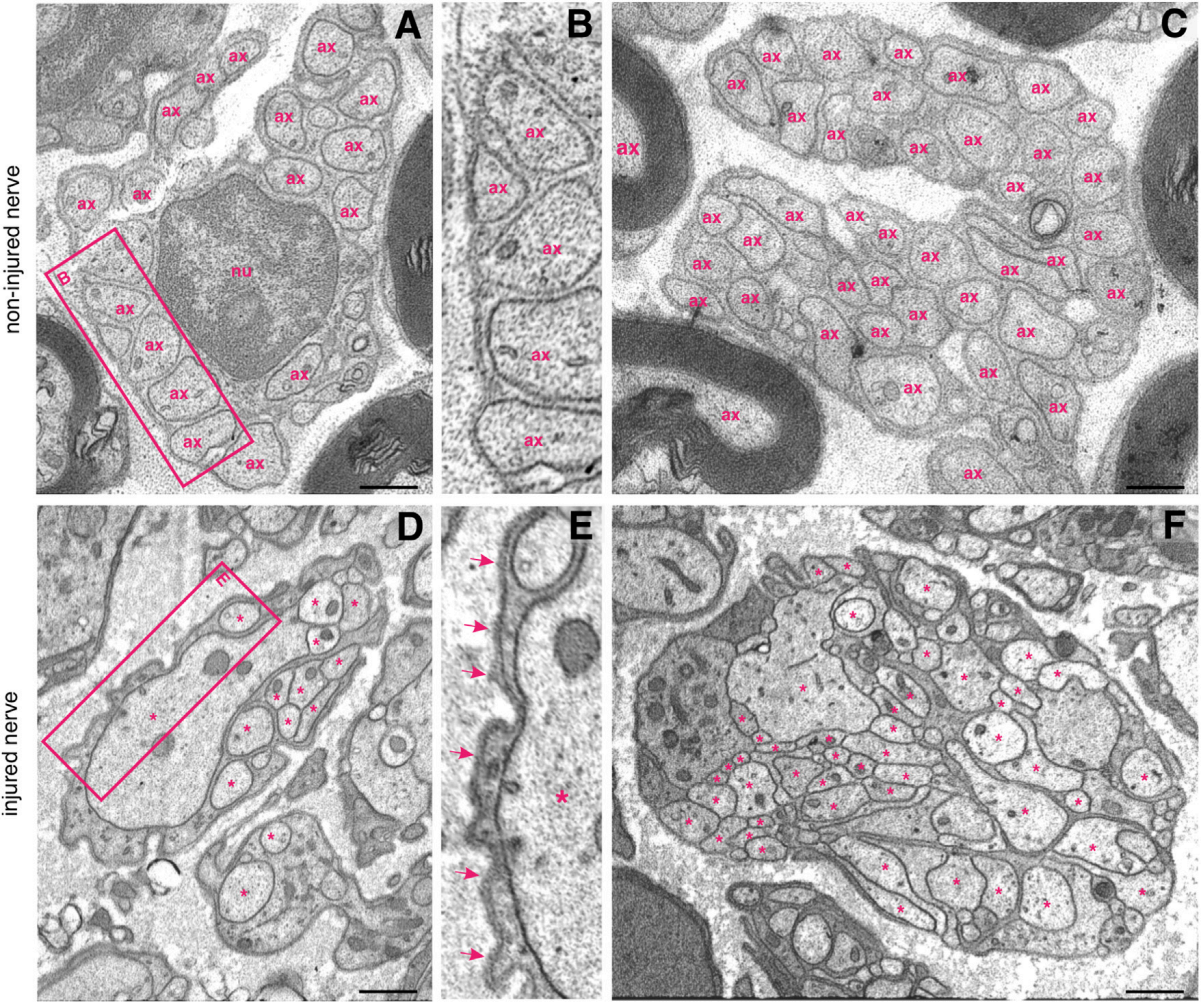
Ultrastructure of non-myelinated fibre bundles in a non–injured nerve and in a regenerating proximal nerve stump. **(A)** Multiple non–myelinated axons (ax) hosted by a non–myelinating Schwann cell. Each axon is well separated from the others **(B)** and more than 40 axons can form a so–called “Remak bundle” **(C)**; nu—nucleus. **(D)** A single non-myelinating Schwann cell uses its lamellipodia to accomodate axonal sprouts of different calibres (asterisks), but no myelination occurs. Note the difference between the Schwann cell basal lamina of the intact **(B)** and an injured nerve **(E)**, arrows indicate the electron-dense basal lamina. **(F)** More than 50 irregularly shaped axonal sprouts (asterisks) can be tightly packed within a non–myelinating Schwann cell. The Schwann cell pseudopodia appear electron–denser than sprouts. Staining: osmium tetroxide, potassium (III) hexacyanoferrate and post–contrasting with lead nitrate and uranyl acetate. Specimen: 55–nm–thin sections of resin–embedded proximal nerve stumps, 10 days after transection performed in C57Bl6/J mice. Scale bar, 500 nm.

**FIGURE 4:**
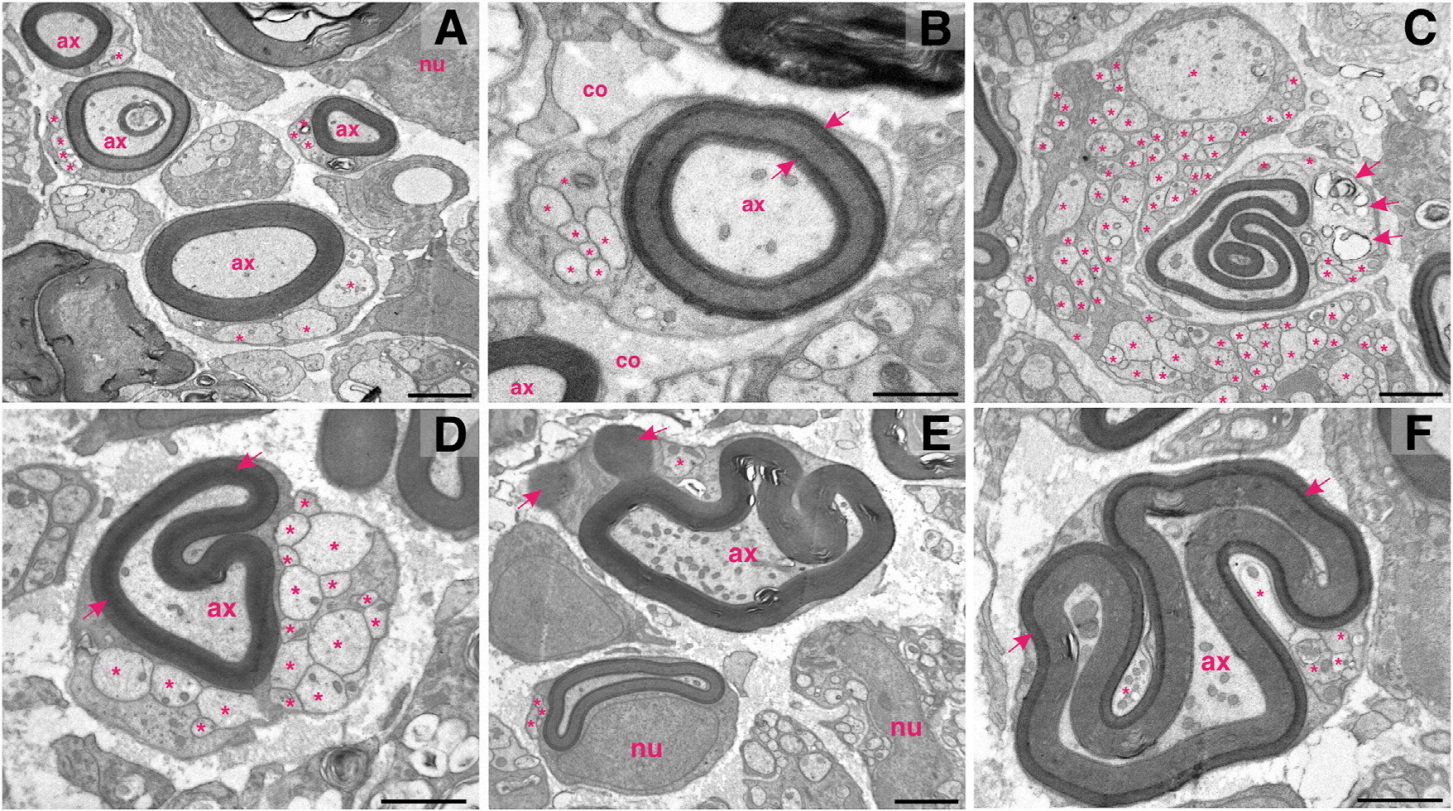
Schwann cells multitasking at the proximal stump of the injured femoral nerve. **(A)** In the field of view: four myelinating Schwann harbouring well–preserved myelin lamellae around the injured axons (ax) and at the same time accommodating axonal sprouts (asterisks). **(B)** The newly formed myelin around the injured axons can vary in its compactness (arrows indicate densely formed lamellae); co—collagen; nu—nucleus. **(C)** Hyperplastic cells: a giant non-myelinating Schwann cells abundant in sprouts (asterisks) adjoining a myelinating Schwann cell. Note that the myelinating Schwann cell contains two separated myelinated axons (one of a remarkable irregularity), a few cytoplasmic sprouts (asterisks) and proteolytic vesicles (arrows). **(D)** While re–shaping the myelin structure and compactness (arrows) around the injured axon (ax), a myelinating Schwann cell can concomitantly host more than 10 axonal sprouts (asterisks). **(E)** A Schwann cell shedding myelin (arrows) while still preserving the axon (ax) and at the same time carrying a sprout (asterisk). Note the abundancy of mitochondria within the axon. In the vicinity: another myelinating and two non-myelinating Schwann cells; nu—nucleus. **(F)** Hyperplasticity in confined space: arrows indicate densely formed myelin lamellae; asterisks highlight sprouts; arrows indicate mylein shedding. Staining: osmium tetroxide, potassium (III) hexacyanoferrate and post-contrasting with lead nitrate and uranyl acetate. Specimen: 55–nm–thin sections of resin–embedded proximal nerve stumps, 10 days after transection performed in C57Bl6/J mice. Scale bar, 1 µm.

The Schwann cells are peripheral glial cells that isolate the axons by forming myelin sheaths (Figure 1), thus increasing the axonal membrane resistance towards a greater action potential propagation velocity. These peripheral glial cells are also involved in the control and guidance of axonal growth and regeneration (Jessen et al., 2015; Jessen and Mirsky, 2019b), demyelination and debris scavenging through autophagy and phagocytosis in the event of Wallerian degeneration (Jang et al., 2016; Wong et al., 2017; Nazareth et al., 2021) (Figures 1–4), maintenance of the micromilieu around an axon and its regulation through reciprocal contacts (Bouçanova and Chrast, 2020), synaptic transmission (Alvarez-Suarez et al., 2020), and immunomodulation (Zhang et al., 2020). Due to their remarkable developmental plasticity (Boerboom et al., 2017; Castelnovo et al., 2017), apart from quiescent myelinating or non–myelinating entities, the Schwann cells represent a very heterogenous population, which can rapidly trans– or de–differentiate into repair–, bridge–, phagocytic or mesenchymal–like phenotypes in the case of nervous system injury (see also Figures 2–4), thus guiding the re–growing axons (Jessen and Mirsky, 2016) to their destination. Multifunctional Schwann cells with myelinating and non–myelinating properties have been found to simultaneously ensheath different axonal types in intact nerves of transgenic mice lacking the Fbxw7 component of the E3 ligase (Harty et al., 2019). Interestingly, similar myelinating/Remak Schwann cell hybrids can be found in regenerating nerves of wild–type mice (Figure 4), suggesting that this morphological transformation is fundamental for regeneration. The ensheathing plasticity of the Schwann cells seems to fluctuate in time and space and has important implications for our understanding of circadian myelination and myelin repair in both, the central and peripheral nervous system.

## Circadian clock in central glia and its role in disease

Pioneering work based on transcriptomic studies in rodents has revealed that many transcripts coding for proteins involved in myelination, synthesis and maintenance of plasma membrane components, e.g. opalin, plasmolipin and Qk, are upregulated in the sleeping brain (Vivo and Bellesi, 2019). In turn, sleep deprivation leads to an upregulation of pro–apoptotic and pro–inflammatory markers such as the apoptotic chromatin condensation inducer 1 (Acin1), heat shock protein family E member 1 (HSPE1), and golgin subfamily A member 3 (GOLGA3), thus indicating an overall beneficial function of sleep for myelin’s maintenance and turnover (Vivo and Bellesi, 2019). Bellesi and colleagues have further shown that the oligodendrocyte progenitor cells have phasic behaviour, namely these cells proliferate during the REM phase of sleep and differentiate during vigilance (Bellesi et al., 2013). However, the mechanisms through which sleep acts in favour of myelination have not been systematically studied, but the neurotransmitters acetylcholine and noradrenaline have been speculated as possible antagonistic regulators of oligodendrocyte progenitor proliferation based on their daytime–dependent secretion modes (Vivo and Bellesi, 2019). Of note, the arousal stimulator noradrenalin, whose concentration in brain peaks during daytime, has been shown to induce oxidative stress and apoptosis in cultured oligodendrocyte progenitors (Khorchid et al., 2002), whereas acetylcholine that is elevated during the wake state and REM sleep, has been shown to promote mitotic expansion of oligodendrocyte progenitors (Paez-Gonzalez et al., 2014; Bellesi, 2015). The observed effects depend possibly on circadian oscillations in the noradrenergic and cholinergic nuclei—in fact, the noradrenalin concentration seems to follow a 24–hour cycle in the prefrontal cortex (Julia M. L. Menon et al., 2019), the noradrenergic *locus caeruleus* neurons have been shown to exhibit a circadian rhythm with a maximum impulse activity during the period of vigilance (González and Aston-Jones, 2006). Analogously, the cholinergic neuronal populations have displayed a rhythm with maximal expression of acetylcholine during the active period (Kametani and Kawamura, 1991; Takase et al., 2007; Takase et al., 2009; Hut and van der Zee, 2011). It is very likely that oscillations of catecholamines co–regulate the circadian clock in oligodendrocytes. Indeed, circadian dysregulation has been associated with myelination disorders in the central nervous system, i.e. enhanced risk of multiple sclerosis correlated with mutations in ARNTL/BMAL1 and CLOCK circadian genes (Lavtar et al., 2018), owing to the regulatory role of the ARNTL gene in oligodendrogenesis (Huang et al., 2020). Patients suffering from multiple sclerosis have had a higher risk of sleeping disorders (Najafi et al., 2013; Tonetti et al., 2019), and an imbalanced melatonin secretion rhythm (Damasceno et al., 2015). The pituitary axis as well as melatonin have been hypothesised to be involved in the pathogenesis of multiple sclerosis, possibly by regulating proliferation and maturation of oligodendrocytes in a circadian manner (Olivier et al., 2009; Ghareghani et al., 2017). Rhythmicity based on the light–dark–cycle (circadian rhythm) is a property exhibited by almost all organisms on Earth. At microscopic scale in form of a cell–autonomous pace, rhythmicity has been documented in hypothalamic neurons of the suprachiasmatic nucleus and somatomotoneurons (Herr et al., 2018), visceromotoneurons (Kelly et al., 2020), astrocytes (Brancaccio et al., 2019), fibroblasts (Hida et al., 2017), myocytes (Perrin et al., 2015), and immune cells (Annamneedi et al., 2021). Various studies have reported cell morphology changes occurring in a circadian manner in neurons (Jasinska et al., 2020) and fibroblasts (Hoyle et al., 2017), and active variations in subcellular morphology, e.g. mitochondrial architecture (Sardon Puig et al., 2018). Both, the circadian cyclicity and cell cycle, have been understood to be intertwined (Johnson, 2010; Masri et al., 2013). Interestingly, fluctuations in the cytosolic calcium concentration in fibroblasts seem to follow oscillations in the transcription levels of clock genes (Wilkaniec et al., 2016). Moreover, circadian rhythmicity has been suggested to underly tissue homeostasis and regeneration (Paatela et al., 2019). In this constellation, we became interested in whether melatonin and circadian rhythm are relevant for peripheral nerve regeneration and homeostasis concerning Schwann cells, the functional relatives of oligodendrocytes.

## Circadian oscillations in peripheral nerve homeostasis

Some physiological parameters of nerve function, such as conduction velocity, may vary during the day or alter upon disruption of the circadian cycle. There are reports stating that the nerve conduction velocity depends on the circadian clock (Ferrario et al., 1980; Montagna et al., 1985). In particular, the sensory fibre conduction velocity has been found to follow a diurnal rhythm (Montagna et al., 1985). According to Ferrario and colleagues, the circadian rhythm in nerve fibre conduction velocities turned out to differ in the sensory and motor fibres (Ferrario et al., 1980). Montagna and colleagues could also observe a circadian trend in the sensory fibre conduction velocity (Montagna et al., 1985). In contrast, the diurnal variations of the motor fibre conduction velocity found by Ferrario and colleagues (Ferrario et al., 1980) could not be reproduced in a recent study (Vishwakarma and Yadav, 2020). These discrepancies might be partially based on differences in sampling, as Vishwakarma and Yadav have employed a larger sample size than Ferrario et al. Interestingly, Maehara and colleagues have concluded that the motor fibre conduction velocity tended to decrease in rats kept at a reverted light–at–night cycle (Maehara et al., 1985). These combined findings reveal that the sensory nerve conduction velocity may follow a circadian trend and/or depend on the circadian cycle. Obviously, the time point of measurement is a critical factor to consider when studying circadian oscillations in peripheral nerve homeostasis.

## Circadian rhythm in peripheral nerve injury

**FIGURE 5:**
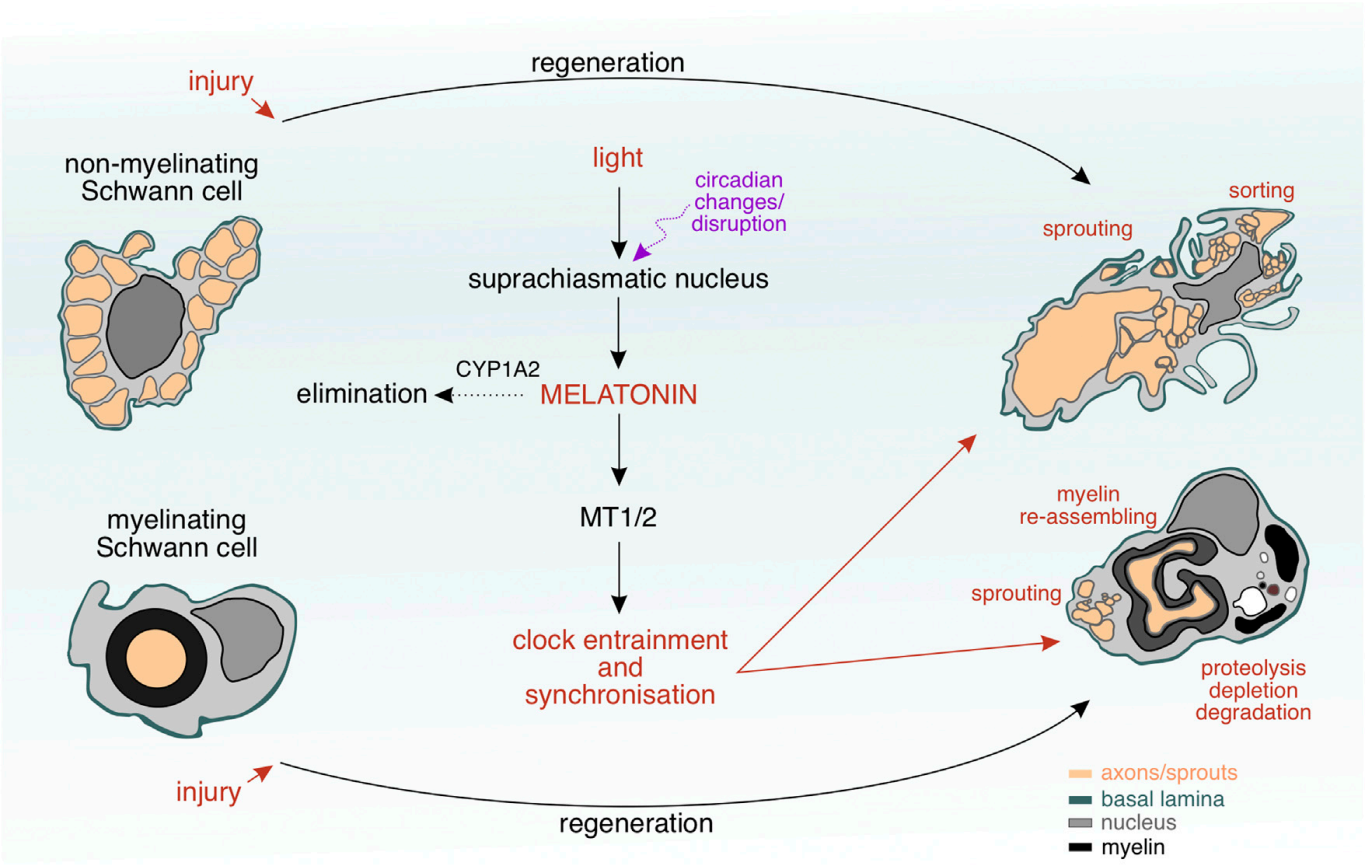
Interplay between Schwann cell plasticity and circadian clock during nerve regeneration. The Schwann cells undergo a remarkable transformation during nerve regeneration upon injury. Variations in light’s intensity due to circadian changes or disruption result in an altered activity of the suprachiasmatic nucles and melatonin concentration levels, to which the Schwann cells can respond selectively *via* their melatonin receptors (MT1/2). Degradation of melatonin by CYP1A2 or changes in blood–nerve barrier’s permeability are regulatory mechanisms allowing a certain amount of melatonin to penetrate the regenerating nervous tissue and to reach the Schwann cells within. MT1/2 may play a role as “chronosensors” to melatonin changes and induce internal clock changes, which allow the Schwann cells to unfold a complex morphology, where multiple events (sprouting, myelin re–assembling, proteolysis, depletion, degradation, and sorting) occur simmulatneously.

The chronobiological component of nerve homeostasis may greatly influence the success of neurorepair therapies. Thus, Zhu and colleagues have demonstrated that daytime therapy employing pulse electromagnetic field has improved sciatic nerve regeneration in rats far better than nocturnal therapy (Zhu et al., 2017). Rateb and colleagues have shown that melatonin treatment has improved peripheral nerve regeneration in rats, in particular, nocturnal treatment with melatonin has led to better results than the daily applications (Rateb et al., 2017). Another study has come to a similar conclusion, when applying melatonin to rats with injured sciatic nerves at night (Moharrami Kasmaie et al., 2019). Kaya and colleagues have shown that disruption of the circadian cycle could influence nerve regeneration—rats which had been kept at a normal 12 h light/dark cycle under treatment with melatonin during daytime have responded to the therapy better than rats which had been kept at inverted light–at–night cyclicity and treated at night (Kaya et al., 2013). In conclusion, external Zeitgeber clues provided by a light source at distinct time points entail different grades of peripheral nerve regeneration. Could this be explained by assuming a circadian rhythm of Schwann cell functions in the process of regeneration? Rateb and colleagues could prove that rats with a sciatic nerve injury under pulsed electromagnetic field therapy had higher number and area of myelinated axons, lower g–ratio and higher S100 expression in the regenerated nerves when treated during daytime compared to night treatments (Rateb et al., 2017). Furthermore, nocturnal application of melatonin has led to an increased myelination and S100 immunoreactivity in crushed sciatic nerves compared to the daily application, whereas there was no difference between both groups in immunoreactivity against the Neurofilament–200 protein (Moharrami Kasmaie et al., 2019). In contrast, no regenerative difference has been observed between animal groups treated with curcumin either during the day or night (Moharrami Kasmaie et al., 2019). Kaya and colleagues have stated that after sciatic nerve injury the density and thickness of myelinated fibres were higher in animals treated with melatonin and kept at a normal illumination cycle than in the animals treated with melatonin and kept at an inverted cycle, yet no quantification and statistical analysis have been provided by the authors (Kaya et al., 2013). It becomes clear that the Schwann cells react differentially to treatment times and circadian rhythm alterations *in vivo* (see Figure 5), and their response may vary depending on the given therapy. Rateb et al. and Moharrami Kasmaie et al. have tried to explain the better results of the nocturnal melatonin application by an additive effect of the endogenous and exogenous melatonin concentrations (Rateb et al., 2017; Moharrami Kasmaie et al., 2019). Since the nocturnal concentration of melatonin is approximately 3–10 times higher than the daily one (Odaci and Kaplan, 2009), the nocturnal application of the exogenous melatonin would be reinforced by the physiological peak of melatonin at night, thus leading to enhanced effects on regeneration according to Rateb et al. and Moharrami Kasmaie et al. (Rateb et al., 2017; Moharrami Kasmaie et al., 2019). However, this explanation becomes incoherent, when one considers the concentration ranges of melatonin application *versus* endogenous melatonin levels. This can be plausibly demonstrated with a few simple calculations.

Following the experimental design from the study of Moharrami Kasmaie et al., 2019, a 200 g rat injected intraperitoneally with 10 mg/kg of melatonin would receive 2 mg of melatonin. If we account for 74% bioavailability of melatonin after an intraperitoneal injection (Yeleswaram et al., 1997), the resorbed amount of melatonin would then be 1.48 mg. According to the formula BV=0.06×BM+0.77 , where BV is the total blood volume and the BM is the body mass of a rat (Lee and Blaufox, 1985), the blood volume of a 200 g rat would be 12.77 ml. Thus, the final concentration of the exogenously applied melatonin in a blood volume of 12.77 ml would be 0.11589 mg/ml or 115.89 × 10^6^ pg/ml. According to Wolden-Hanson and colleagues, the diurnal physiological melatonin concentration in rat serum is 20 pg/ml, at night it rises to 50 pg/ml (Wolden-Hanson et al., 2000). Hence, the total nocturnal concentration of exogenous and endogenous melatonin (115.89 × 10^6^ pg/ml + 50 pg/ml) compared to the total concentration of diurnal endogenous and exogenous melatonin (115.89 × 10^6^ pg/ml + 20 pg/ml) would negligibly deviate by 2.58 × 10^−5^%. Based on these calculations, we assume that at the given microgram range of applied melatonin the contribution of the pico–range variability provided by the physiological oscillations of endogenous melatonin is minimal. Therefore, this negligible additive effect cannot lead to the observed differences between neuroregeneration upon nocturnal and diurnal melatonin application. What is the reason for an increased responsiveness of regenerating nerves to melatonin treatment at night? A plausible argument is the circadian variation of melatonin degradation in the body. Elimination of melatonin occurs mostly through hepatic metabolisation with cytochrome P450 enzyme CYP1A2 (see also Figure 5), followed by glucuronidation or sulfatidation (Tordjman et al., 2017). In fact, the CYP1A2–mediated turnover has a circadian trend (Chen et al., 2020b)—in humans, CYP1A2 activity is minimal in the evening (Perera et al., 2013). This and other studies have proposed that expression of some hepatic cytochrome P450 enzymes is controlled by the circadian clock (Lu et al., 2013; Zhang T. et al., 2018; Deng et al., 2018; Lin et al., 2019).

An alternative school of thought considers that the permeability of the perineurium may increase at night and allow more melatonin to penetrate into the neural interstitium, where the hormone can unfold its pro–regenerative effects. There is an indication that nerve permeability depends on the circadian cycle (Maehara et al., 1985). The authors have studied permeability changes in peripheral nerves of rats kept in normal vs reversed light–dark cycle (Maehara et al., 1985). Curiously, the animals kept at an inverted cycle, when treated with lead acetate, accumulated more lead in their nerves than the group under normal illumination cycle (Maehara et al., 1985). These findings imply a variable permeability of the blood–nerve barrier throughout the day. Furthermore, permeability variations have been reported also for the blood–brain barrier (Zhang S. L. et al., 2018; Cuddapah et al., 2019; Zhang et al., 2021). Hence, chronopharmacology may become an important factor in clinical practice, when optimizing drug application schedules related to times at which the particular drug can cross the blood–nerve barrier, thus yielding optimal therapeutic outcome. Interestingly, in patients suffering from neuropathic pain the pain intensity tends to maximise in the evening (Gilron et al., 2013). Moreover, peripheral diabetic neuropathy seems to occur more often in equatorial, subequatorial or tropical countries (e.g. Egypt, Saudi Arabia), when compared to locations with more temperate climate, restricted illumination, and shorter days in northern latitudes (e.g. United Kingdom, Germany, Belgium) (Azmi et al., 2019). The link between circadian rhythmicity and neuropathic conditions suggests that external light clock variations and time can influence nerve physiology and repair, thus pushing the frontiers towards precision in treatment.

However, there are still considerable limitations of existing data on the chronopharmacological component of nerve regeneration therapy and further in–depth studies need to be carried out. Key experiments establishing a relevant connection between circadian rhythm and peripheral nervous system repair after injury would be feasible in mouse models deficient in major clock genes such as PER, CRY or BMAL1. Of note, enhanced risk of multiple sclerosis has been associated with variations in the circadian genes ARNTL/BMAL1 and CLOCK (Lavtar et al., 2018), likely due to the regulatory role of the ARNTL gene in oligodendrogenesis (Huang et al., 2020). Therefore, one could expect that clock–gene mutations may influence the myelination process by Schwann cells in the periphery as well. Unfortunately, there is a gap in the literature concerning peripheral nerve regeneration in clock gene knock–out mouse strains. The comparison between the native nerve ultrastructure in such mouse strains vs wild–type controls in combination with genome–wide association studies would be an essential step towards closing this gap.

## Does an intrinsic circadian rhythm indwell the Schwann cells?

The first published observations indicating that Schwann cells may possess an intrinsic rhythm were made in 1959. In a time lapse–cinematographic study, cultured Schwann cells have been observed to contract rhythmically approximately every 4 min (Pomerat, 1959). Several other authors have also documented Schwann cell motility in monocultures, acutely dissected nerves, and dorsal root ganglion explants (Ernyei and Young, 1966; Cravioto and Lockwood, 1968; Dubois-Dalcq et al., 1981; Forman et al., 1986). The cell undulations have been relatively constant in duration, although the intervals between the undulations had varied between 2 and 18 min, thus lacking any stringent periodicity (Ernyei and Young, 1966; Cravioto and Lockwood, 1968; Dubois-Dalcq et al., 1981; Forman et al., 1986). It has been found that the time point of movement and intervals between undulations depend on the culturing conditions for Schwann cells (Dubois-Dalcq et al., 1981; Forman et al., 1986). Dubois-Dalcq and colleagues have further confirmed that Schwann cell undulations are relatively constant in duration and of a certain nature as compared to sporadic perineural fibroblast movements (Dubois-Dalcq et al., 1981). In spite of the apparent lack of synchronicity between the cells and the variable periodicity between the Schwann cell undulations, the movement duration and the number of movements per unit of time had remained rigorously constant (Dubois-Dalcq et al., 1981). Similarly, Forman and colleagues have also noted that regardless of the culturing media, the distribution of Schwann cell pulse durations over time had stayed constant (Forman et al., 1986). It is important to mention in this respect that the cell culture is a simplistic model and lacks therefore oscillating humoral components of the living organism affecting the cellular clock, such as melatonin. In addition to that, minimal interference from variables such as unwanted light contamination, pH and medium osmolarity shift, or cell confluency may lead to cell clock dyssynchronisation (Beaulé et al., 2011). Differences in sampling and selection of the particular cell entity may also lead to variable results. For instance, cultured suprachiasmatic neurons are still able to express clock genes (Beaulé et al., 2011), whereas other cell types such as NIH–3T3 cells and astrocytes may dampen their circadian properties over the time in culture and require co–cultured suprachiasmatic slices to re–activate their clock (Prolo et al., 2005; Li et al., 2008). Perhaps, similar conditions are required for cultured Schwann cells to maintain or re–wind their circadian clock.

An indirect evidence for an intrinsic cycle in Schwann cells has come from studies on bone marrow reorganisation revealing the non–myelinating Schwann cells as regulators of the hematopoietic stem cell activity by keeping the hematopoietic stem cells in a hibernating state, from which they can switch to active proliferation, for example, after a haemorrhage (Yamazaki et al., 2011). Considering the fact that a portion of stem cells in the bone marrow remains dormant and awakens, only if necessary, around every 145 days according to simulations by Wilson and colleagues (Wilson et al., 2008), this phenomenon of switching between active and dormant state resembles an infradian cycle that could perhaps be controlled by a similar cycle in Schwann cells. It has been also reasonably assumed that light intensity changes in terms of photoperiodicity may have a direct impact on Schwann cells’ activity. According to Vera and colleagues, however, the Schwann cells do not seem to possess a light–sensitive protein machinery that could influence them, since light irradiation of Schwann cells had not led to any morphological, proliferatory or metabolic alterations (Diaz Vera et al., 2021). Intriguing findings have been described in a chemotherapy–induced peripheral neuropathy model, as according to Kim and colleagues the mechanical threshold in paclitaxel–induced neuropathic pain has shown circadian oscillations in rats, with a minimal pain tolerance observed during the inactivity phase (daytime for rats). Furthermore, the authors have reported oscillating expression levels of the key clock genes BMAL1 and PER2 in satellite cells from cultured dorsal root ganglia explants, with PER2 being expressed stronger in satellite cells than in neurons (Kim et al., 2020). These results imply that satellite glial cells have a circadian clock. Taking into account reported similarities in the transcriptomic landscape, morphology and shared ontogenetic origin from the neural crest (Jessen and Mirsky, 2019a; Milosavljević et al., 2021), the ganglionic satellite glial cells have been hypothesised to represent an arrested developmental stage within the Schwann cell lineage (George et al., 2018). This is further supported by the finding that ganglionic satellite cells can differentiate into Schwann cells (Sundaram et al., 2021). Several attempts have been made to transcriptionally profile the Schwann cells (Franssen et al., 2008; Ulrich et al., 2014; Arthur-Farraj et al., 2017; He et al., 2018; Silva et al., 2019; Chen et al., 2021; Brosius Lutz et al., 2022). According to the transcriptional profiling of The Sciatic Nerve Atlas by Gerber and colleagues, single–cell RNA sequencing has revealed that Schwann cells express PER1, PER2, PER3, CRY, CRY2, ARNTL, and ARNTL2 genes in a dynamic manner over the course of the early postnatal development (Gerber et al., 2021). Obviously, the Schwann cells can respond to external clock input changes (see Figure 5) and the collective experimental evidence implies that the Schwann cells might be equipped with a “time–sensitive protein machinery.” The melatonin receptors can be sensors for melatonin oscillations upon external clock changes. However, further investigations are necessary to test this hypothesis.

## Melatonin’s effects on neuroregeneration

Recent studies using various lesion models of the central and peripheral nervous system have indicated melatonin–induced neuroprotective effects occurring in a dose–dependent manner and unfolding their full magnitude at supraphysiological concentrations around 50 mg/kg in a (systemic) long–term administration (Gül et al., 2005; Shokouhi et al., 2008; Atik et al., 2011; Li et al., 2014). Systemic intraperitoneal administration of melatonin in mice after facial nerve injury has been shown to preserve the thickness of myelin sheaths, whereas given electrophysiological parameters of nerve conduction that included the latency and amplitude of the compound muscle action potentials have remained unaltered (Tuna Edizer et al., 2019). Edizer and colleagues have also observed reduced immunohistochemical reactivity to collagen III and V in the treated nerve as well as minimal immune infiltration and normal structure of axons and neurofilaments (Tuna Edizer et al., 2019); for more details see Table 1. Of note, also topically applied melatonin has been found to support regeneration, yet not at the same level of magnitude as to when the hormone had been administered systemically (Tuna Edizer et al., 2019). It is noteworthy in this context that subcutaneous melatonin injection has been found to significantly improve Schwann cell proliferation and migration as well (Pan et al., 2021). Similar histomorphological findings were obtained when applying melatonin to injured nerves in other studies, but the electrophysiological parameters contradicted the data of Edizer and colleagues. Atik and collaborators have observed the occurrence of a thinner epineurium and better organisation of the reduced endoneural collagen at the proximal stump of the dissected sciatic nerve, less distinctive de–myelination and loss of axons distally; additionally, the authors have recorded shorter compound muscle action potential latencies, higher amplitudes and conduction velocities of the axion potentials in a damaged nerve treated with melatonin when compared to the control group (Atik et al., 2011). Similarly, Qian and colleagues have described higher nerve conduction velocities and improved morphological parameters (thicker myelin sheaths, higher regenerated axonal area, myelinated axon number and diameter) in rats treated with an implanted melatonin/polycaprone nerve guidance conduit in comparison to rats that had received a polycaprone implant without melatonin (Qian et al., 2018). Guo and colleagues have found shorter compound muscle action potential latencies when treating rats with melatonin after brachial plexus nerve–root avulsion injury—the motoneuron survival rate and motor endplate regeneration was enhanced (Guo et al., 2019). Other scientists have observed fewer neuromas in rats treated with melatonin following a neurorrhaphy of transected sciatic nerves (Turgut et al., 2005). Surprisingly, an elevated collagen content and macroscopic neuroma formation were detected in injured sciatic nerves of pinealectomised rats, demonstrating that melatonin deficiency hinders an adequate nerve regeneration, whereas exogenously supplied melatonin alleviated the described effects (Turgut et al., 2005). Further studies have also determined that melatonin improves neuroregeneration in rats after a sciatic nerve lesion (Kaya et al., 2013; Rateb et al., 2017; Moharrami Kasmaie et al., 2019). However, in the majority of reported studies melatonin has been applied systemically, which may cause global and complex effects making conclusion on melatonin–promoted regeneration difficult. Hence, local application of melatonin to peripheral nerves, i.e., *via* intraneural injections or polymer tube nerve reconstruction grafts filled with the therapeutic substance (see Jakovcevski et al., 2022), deems to be a more appropriate application method. Indeed, Zhang and colleagues have applied locally melatonin into the lesioned area of the injured sciatic nerves and found improved myelination and regeneration (Zhang et al., 2022).

**TABLE 1:** Effects of melatonin on Schwann cells in the scenario of nerve regeneration.

*General effects on Schwann cells*		
Event	Effect	References
Proliferation	↑	Chang et al. (2014); Tiong et al. (2020); Pan et al. (2021); Govindasamy et al. (2022)
De–differentiation	↑	(Tiong et al. (2020); Govindasamy et al. (2022)
Migration	↑	Pan et al. (2021)
Extracellular matrix		
Collagen	↓	Atik et al. (2011); Tuna Edizer et al. (2019)
Matrix metalloproteinases	?	
Chondroitin sulfate proteoglycans	↓	Krityakiarana et al. (2016)
FAK, p-FAK(Tyr576/577, Tyr397)	↑	Govindasamy et al. (2022)
RedOx status and inflammation		
SOD, catalase, GPX	↑	Sayan et al. (2004); Chang et al. (2008); Erol et al. (2008)
NO–synthase	↓	Uyanikgil et al. (2017)
TNF–α, IL–1β, IL–6	↓	Rateb et al. (2017)
Transcription and signalling		
MT1	↑ (RT4 cells treated with 1–5 μM melatonin, injured animals treated with 1 and 10 mg/kg melatonin)	Chang et al. (2014); Tiong et al. (2020)
MT1, MT2	↓ (RT4 cells treated with 10 μM melatonin)	Tiong et al. (2020)
GDNF	↑	Tiong et al. (2020)
SHH, Gli1	↑	Pan et al. (2021)
BDNF	↑	Hashimoto et al. (2008)
Ras, p–B–Raf, p–C–Raf	↑	Tiong et al. (2020)
p–SAPK–JNK(p54)/SAPK–JNK(p54); p–SAPK–JNK(p46)/SAPK–JNK(p46); p–p38/p38	↓/↑; ↓/↑; ↓/↑	
p–ERK(p44)/ERK(p44); p–ERK(p42)/ERK(p42)	↑/↓; ↑/↓	Chang et al. (2014); Tiong et al. (2020); Stazi et al. (2021)
SOX2	↑	Tiong et al. (2020); Govindasamy et al. (2022)
pNF-κB	↑	Govindasamy et al. (2022)
IKK-α	↑	

↑(upregulation), ↓ (downregulation), = (no effect), ? (unclear).

Could the neuroregeneration promoting effects of melatonin be reinforced if the hormone were to be used as a component of a complex therapy combining multiple drugs? In this respect, some work groups have reported that peripheral nerve injury treatment is more efficient, when melatonin had been combined with other substances. For example, melatonin and chondroitin sulfate ABC have maximised the positive outcome in rats after a brachial plexus nerve–root avulsion injury, when compared to single melatonin or chondroitin sulfate ABC therapy (Guo et al., 2019). Chen and colleagues have proposed to use melatonin coupled to magnetite (Fe_3_O_4_) nanobeads for a controlled sequential drug release, thereby relying on the finding that pure Fe_3_O_4_ nanobeads had promoted peripheral nerve regeneration (Chen X. et al., 2020). Zhang and colleagues have used combined melatonin with adipose tissue–derived stem cells, and showed that this combined therapy delivers better results than therapy with melatonin alone (Zhang et al., 2022). However, it remains unclear whether such a combined melatonin therapy would be an option for human patients. Still, we are not able to predict how drug interactions would influence the regenerative outcome, regarding the scare literature dealing with such topics.

Considering the effects of melatonin on the expression of various collagen types in injured nerves, we next raised the question of how melatonin might influence reorganisation of the extracellular matrix and perhaps the basal lamina around the Schwann cells.

## Melatonin modulates the extracellular matrix composition and may regulate assembling of Schwann cells’ basal lamina

The findings that melatonin downregulates collagen in injured nerves (Turgut et al., 2005; Atik et al., 2011; Tuna Edizer et al., 2019) lead to the conclusion that melatonin affects the reorganisation of the extracellular matrix (ECM), see also Table 1. The ECM is a scaffold that gives each tissue the mechanical properties of elasticity and solidity (Rosso et al., 2014; Belin et al., 2017), and creates a microenvironment that influences the intercellular connectivity and communication (Kim et al., 2011; Valiente-Alandi et al., 2016; Sapir and Tzlil, 2017), the integration of new cells into the tissue and their adhesion (McMillen and Holley, 2015; Gkretsi and Stylianopoulos, 2018), and co–determines the fate of cell differentiation and function (Sainio and Järveläinen, 2020). Further tasks of the ECM in the nervous system include formation of Ranvier nodes (Frischknecht et al., 2014), control of neural plasticity (Frischknecht et al., 2014; Dzyubenko et al., 2016), assistance in axonal pathfinding and guidance (Roumazeilles et al., 2018), myelination (Eldridge et al., 1989), and development of the neuromuscular junctions (Chan et al., 2020). Moreover, the ECM modulates the activity of Schwann cells (Chernousov et al., 2008) and axonal growth (Myers et al., 2011; Song and Dityatev, 2018). One of the Schwann cell organelles, the Golgi apparatus, plays a crucial role in production of a major part of the ECM proteins. The cell organelle provides specific post–translational modifications and guides the substances by means of vesicular transport to secrete them into the interstitial space (Unlu et al., 2014; Hellicar et al., 2022).

Collagen is an abundant component of the ECM—the molecule is important for formation of the ECM, as in absence of collagen, ECM assembly is impaired, and this in turn, disrupts the myelination process (Podratz et al., 2001; Mouw et al., 2014). Several studies have described that the Schwann cells produce collagen type V, which inhibits axonal outgrowth on the one hand, but promotes Schwann cell migration and association with axonal sprouts on the other (Chernousov et al., 2000; Chernousov et al., 2001). Vitale and colleagues have reported that Schwann cells express collagen type VI, while differentiating into mature myelinating phenotypes, thus being no longer dependent on neuregulin (Vitale et al., 2001). Subsequent investigations have unveiled that collagen VI regulates peripheral myelination by setting a limit to the thickness of the myelin sheaths, especially regarding the fact that collagen VI knock–out mice have displayed hypermyelination (Chen P. et al., 2014). Of note, artificial ECMs, including the collagen–based ones, have been employed to promote guided nerve regeneration (Georgiou et al., 2013; Gu et al., 2014). These collective findings suggest that the interplay between the Schwann cells and ECM is essential for neuroregeneration.

Could melatonin regulate this interplay and change the chemical and biomechanical properties of the ECM? If true, such an assumption may partially explain the effects of melatonin on neurorepair. A number of studies from recent years have shown repeatedly that melatonin reduces dramatically the expression of collagen, and thus, glial scar formation in an injured nerve, thereby enabling the successful neurite regrowth and consecutive muscular reinnervation (Turgut et al., 2005; Atik et al., 2011; Tuna Edizer et al., 2019). Interestingly, extraneural alterations in the production of ECM under the influence of melatonin have been described in other tissues—melatonin has turned out to upregulate the levels of collagen II, collagen X and aggrecan in chondrogenic mesenchymal stem cells (Gao et al., 2014), and increase the expression of glycoproteins, fibronectin and laminin in several cancer cell lines (Sung et al., 2016). However, melatonin treatment has reduced the production of collagen, hydroxyproline, laminin and hyaluronan in hepatic tissue, thus alleviating tetrachlormethane–induced fibrosis (Hong et al., 2009) and tissue scarring in pinealectomised rats (Drobnik and Dabrowski, 1996).

Melatonin seems to affect the composition of the ECM also by modulating the expression of proteases. Although hardly detectable in tissue, expression and activation of the matrix metalloproteases (MMPs) begins upon injury of the nervous system (Bellayr et al., 2009). The MMPs destroy the blood–nerve and blood–brain barrier, cleave and remodel the proteins of the ECM to allow a higher grade of permissibility for inflammatory cells, and modulate nociception (Shubayev et al., 2006; Reinhard et al., 2015; Rempe et al., 2016; Zhao et al., 2020; Deng et al., 2021). MMPs have been shown to control proliferation and phenotypic status (Chattopadhyay and Shubayev, 2009; Kim et al., 2012; Lu et al., 2020), migration (Mantuano et al., 2008) and myelination behaviour (Wang et al., 2019) of Schwann cells. In injured nerves, MMP–2 (gelatinase A, or 72 kDa type IV collagenase), MMP–3 (stromelysin–1) and MMP–9 (gelatinase B, or 92 kDa type IV collagenase) have been found upregulated (La Fleur et al., 1996; Kherif et al., 1998; Remacle et al., 2018), and the Schwann cells were proven to be the source of MMPs (Chattopadhyay and Shubayev, 2009; Oliveira et al., 2010). Additionally, the tissue inhibitor of metalloproteinase 1 (TIMP–1), which has been speculated to act as a protective agent for the basal membrane of Schwann cells, has been found upregulated in injured nerves (La Fleur et al., 1996). Liu and colleagues have also shown that nerve injury induces expression of TIMP–1 and activation of spinal glia and suggested that short–term inhibition of proteolysis through TIMP–1 enables growth of axons into the central nervous system at Redlich–Obersteiner’s zone (Liu et al., 2015).

However, melatonin seems to predominantly inhibit proteases rather than activate them. For instance, melatonin has been demonstrated to inhibit MMP–9 in a mouse model of cerebral ischemia (Tai et al., 2010). Qin and colleagues have shown that melatonin protects the blood–brain barrier through upregulation of TIMP–1 in pericytes and regulation of the NOTCH/NF–κB pathway (Qin et al., 2019). Furthermore, melatonin has reduced the expression of MMP–9 in a human gastric adenocarcinoma cell line and another mechanism of MMP–inhibition through a direct interaction of melatonin with Pro421 and Ala191 at the catalytic centre of MMP–9 has been found (Rudra et al., 2013). Melatonin has been shown to also inhibit MMP–13 in prostatic cancer, thereby possibly attenuating metastasis (Wang et al., 2021). The hormone has also stimulated release of the tissue factor pathway inhibitor from the endothelium (Kostovski et al., 2011), and suppressed the proteolytic cascades of blood coagulation, thus reducing the risk of thrombosis (Wirtz et al., 2008). Melatonin has stimulated the proteolytic cleavage of β–amyloid precursor protein with A Disintegrin And Metalloproteinase domain 10 and 17 (ADAM 10, ADAM 17).

In peripheral nerves, the effects of melatonin on MMP’s expression and activity have not been studied so far. Melatonin has been proven to downregulate chondroitin sulfate proteoglycans after spinal cord injury (Krityakiarana et al., 2016). Chondroitin sulfate proteoglycans are known for their inhibitory effects on neurite growth (Jin et al., 2018), and their degradation unmasks the Schwann cell’s basal lamina and promotes axo–glial interactions (Kuffler et al., 2009). Treatment with MMPs has resulted in degradation of chondroitin sulfate proteoglycans and unmasking the laminin of the Schwann cells’ basal lamina to allow for axo–glial interactions (Kuffler et al., 2009). In this context, one could assume that degradation of chondroitin sulfate proteoglycans in melatonin–treated nerves (Krityakiarana et al., 2016) could depend on activation of the proteases with melatonin, in contrast to other tissue types where melatonin inhibits the MMPs. However, melatonin seems to inhibit proteases’ activity across the entire organism, as it was discussed in the previous paragraph.

In summary, melatonin clearly alters the composition and biomechanical properties of the ECM, thus making the peripheral nerve tissue less stiff and enabling regenerating axons to invade the endoneurium and to reinnervate their targets. Since the Schwann cell basal lamina also belongs to the extracellular space and consists of similar components as the endoneurium, it is a possible scenario that the basal lamina is altered in the Schwann cells exposed to melatonin. The Schwann cells produce the components of their own basal lamina, including different types of collagen (Chernousov et al., 2000; Chernousov et al., 2001; Vitale et al., 2001), NCAM, α2–, α6–, α7–, β1–, and β4–integrins (Roche et al., 1997; Tsiper and Yurchenco, 2002), α2–, α4–, and α5–laminins, β–dystroglycan (Previtali et al., 2003), and L1 (Wood et al., 1990). However, some elements of Schwann cells’ basal lamina are not expressed by the cells themselves, despite the pericellular localisation of those ECM molecules, but rather the fibroblasts of the perineural sheath contribute to the formation of the basal lamina as well (Obremski et al., 1993). Moreover, the molecular composition of the basal lamina is a determinant for Schwann cells’ development and axo–glial interaction in radial sorting and myelination (Wood et al., 1990; Obremski et al., 1993; Court et al., 2006; Urbanski et al., 2016). The basal lamina is important for Schwann cell contractility and spreading, and defects in the basal lamina can affect the differentiation and proliferation of Schwann cells (Grove et al., 2007; Grove and Brophy, 2014). Although the expression of collagen changes in the regenerating peripheral nerves, it is not always a component of the basal lamina, but rather a constituent of the endoneurium. Whether the ultrastructure of Schwann cells’ basal lamina is changed in the regenerating nerve, has not been reported yet. However, this does not rule out the possibility that melatonin can influence the composition of the basal lamina. Interestingly, according to Govindasamy and colleagues, melatonin could regulate the focal adhesion kinase (FAK) in Schwannoma cells and thereby control their proliferation and differentiation status (Govindasamy et al., 2022). FAK can interact with laminins in Schwann cells’ basal lamina (Chernousov et al., 2008). The kinase is alos involved in the radial sorting process (Grove et al., 2007; Grove and Brophy, 2014). FAK–deficiency has led to reduced motility and spreading of the Schwann cells during regeneration and therefore, inability to ensheath axons, which resulted in premature differentiation of the Schwann cells (Grove and Brophy, 2014). However, in adult FAK knock–out mice the basal lamina was formed normally, indicating that FAK function is dispensable for the mature differentiated Schwann cells (Grove and Brophy, 2014). It seems that FAK function is essential only during development or regeneration, when the Schwann cells de–differentiate and resemble an immuature phenotype. Could melatonin integrate into the neuroregeneration process and regulate the basal lamina/Schwann cell contact *via* FAK, thereby supporting remyelination? An important aspect of the regenerative process is the disintegration of injured tissue. This event is accompanied by inflammation and oxidative stress. Could melatonin affect these processes?

## Anti–oxidative and anti–inflammatory effects of melatonin on neuroregeneration

The pro–regenerative potential of melatonin depends partially on its antioxidative (Reiter et al., 2016) and immunomodulatory effects (Hardeland, 2018). Melatonin acts *via* distinct mechanisms, which include the inhibition of oxidative and activation of anti–oxidative enzymes; e.g. melatonin induces an upregulation of the superoxide dismutase (SOD), catalase and glutathione peroxidase (GPX) in peripheral nerves (Sayan et al., 2004; Chang et al., 2008; Erol et al., 2008) and the central nervous system (Limón-Pacheco and Gonsebatt, 2010; Pandi-Perumal et al., 2013). Additionally, the hormone inhibits the NO–synthase in peripheral nerves (Uyanikgil et al., 2017) and reduces the expression levels of the pro–inflammatory cytokines TNF–α, IL–1β, IL–6 in peripheral nerves (Rateb et al., 2017), the central nervous system (Haddadi and Fardid, 2015) or systemically in the serum (Sánchez-López et al., 2018). However, in an experimental model of encephalomyelitis, melatonin has been shown to upregulate expression of IL–10 (Chen et al., 2016) and could prevent inflammatory response in the muscle tissue after exhaustive exercises (Beck et al., 2015). The above mentioned anti–oxidative enzymes are involved in the scavenging of free radicals (Gałecka et al., 2008) inducing oxidative damage in mitochondria (Guo et al., 2013), peroxidation of proteins and lipids in the biomembranes and also specifically in the myelin sheaths (Ravera et al., 2015), protein aggregation, and to a total breakdown of the cellular function (Höhn et al., 2014). The antioxidative effects of melatonin in nerve injury may depend on the expression of parkin, which stimulates mitophagy, thus reducing the generation of reactive oxygen species or radicals (Li et al., 2022).

Melatonin has been found to alleviate mitochondrial dysfunction and to preserve myelin by supporting remyelination in an experimental model of multiple sclerosis (Kashani et al., 2014). The hormone has also prevented mitochondrial damage induced by oxidative stress in hyperglycaemic conditions through upregulation of the antiapoptotic marker Bcl2, and upregulation of NF–κB, mammalian target of rapamycin (mTOR) and Wingless–related integration site (Wnt) pathways in Schwann cells (Tiong et al., 2019). Additionally, melatonin has acted radioprotectively (Farhood et al., 2019). Therefore, melatonin’s employment in therapy of radiation–induced peripheral neuropathy has been speculated as a method of choice for oncopatients (Dheyauldeen et al., 2019). According to those data, melatonin could improve sciatic nerve conduction parameters, elevate total protein concentration and upregulate the SOD and catalase’s activity in rat sciatic nerves when injected intraperitoneally after irradiation.

In summary, melatonin prevents oxidative stress–induced damage in nerves and preserves the myelin in case of exposure to radiation, re–perfusion trauma following ischemia or inflammatory response. This effect is based on the free radical scavenging activity of melatonin through stimulation of antioxidative enzymes, whose chaperone–like functions allow to preserve the structural and functional integrity of proteins and lipids in the biomembranes and cytosol. The described events demand a precise signalling control provided by the melatonin receptors.

## Melatonin membrane receptor signalling in Schwann cells

**FIGURE 6:**
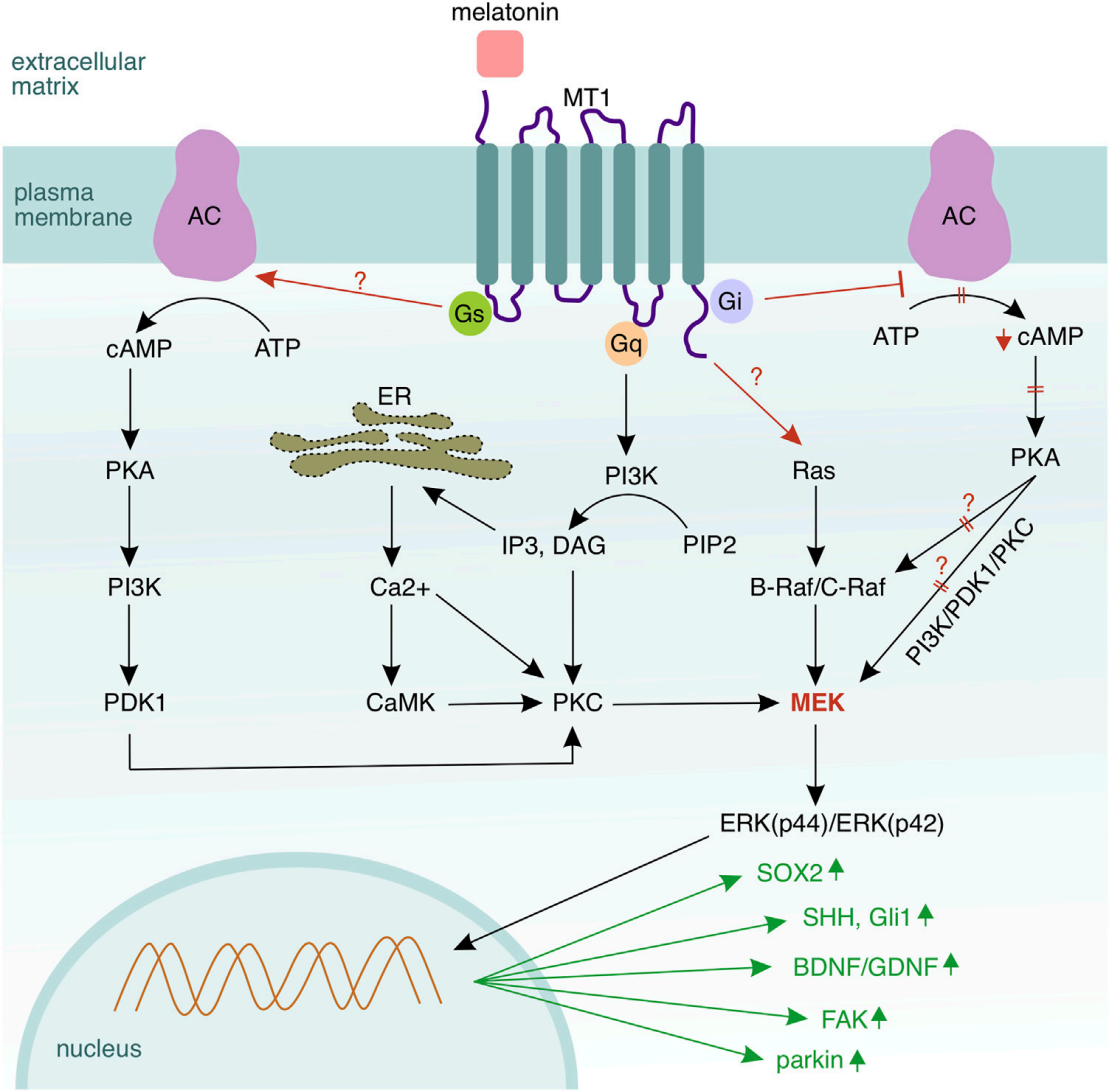
Melatonin signalling in Schwann cells. MT1 is the dominant receptor mediating melatonin signalling in Schwann cells *via* three coupled G–proteins—Gi, Gs, and Gq, and also the Ras/Raf/MEK/ERK cascade. Activation of Gs leads to a conversion of adenosine triphosphate (ATP) to cyclic adenosine monophosphate (cAMP) by the adenylyl cyclase (AC). In turn, cAMP activates protein kinase A (PKA) to phosphorylate mitogen activated protein kinase (MEK) and Raf. MEK activation *via* Gs occurs through sequential phosphorylation of multiple kinases—beginning with cAMP, followed by PKA, phosphatidylinositol–3–kinase (PI3K), phosphoinositide–dependent kinase 1 (PDK1), PKC, and MEK. Activation of Gs inhibits the adenylyl cyclase and the corresponding downstream signalling. Gq, which is coupled to the MT1 receptor, activates phospholipase c (PLC) and hydrolyses membrane–located phosphatidylinositol–4,5–biphosphate (PIP2) to diacylglycerol (DAG) and inositol–1,4,5–triphosphate (IP3). IP3 activates a calcium channel in the endoplasmic reticulum and induces calcium release into the cytosol. DAG and calcium ions synergistically activate protein kinase C (PKC), which can regulate also MEK. In summary, MEK seems to be the central hub of the melatonin–mediated signalling, where all regulatory pathways converge. However, the upstream kinase of the MAPK cascade Ras becomes also activated by the MT1 receptor (the mechanism is not fully clear). Melatonin upregulates SOX2 (Schwann cell de–differentiation), SHH and Gli1 (signalling components of the Sonic hedgehog pathway mediating Schwann cell migration), as well as BDNF and GDNF (brain and glial derived neurotrophic factors supporting the axo–glial interaction). Melatonin elevates the expression of parkin, which mediates mitophagy in Schwann cells, thus reducing the amount of reactive species as well as oxidative stress. Collagen and chondroitin sulfate proteoglycans, which are components of the extracellular matrix, are being downregulated, while the focal adhesion kinase (FAK) that regulates the interplay between a Schwann cell and its basal lamina is upregulated.

Two types of melatonin membrane receptors, MT1 and MT2, have been discovered in mammals (Dubocovich and Markowska, 2005). Chang and colleagues have stated that the Schwann cells express both melatonin receptors, with MT1 expression higher than MT2 in cultured Schwann cells isolated from rat musculocutaneous nerves and RSC96 cells (Chang et al., 2014). Immortalised RT4–D6P2T rat Schwann cells have been shown to express MT1 and MT2 receptors at similar levels (Tiong et al., 2020). Schwann cells have reacted to melatonin treatment at low concentrations with increased MT1 expression, thus suggesting a dominating role of MT1 over MT2 in proliferation and de–differentiation (Chang et al., 2014). Contrariwise, high–dosed melatonin has suppressed the expression of both receptors (Tiong et al., 2020). Stazi and colleagues have also shown that the MT1 expression rises in the Schwann cells and decreases in the axons upon sciatic nerve injury, indicating that the MT1 receptor may be more relevant than the MT2 isoform in neurorepair mediated by melatonin (Stazi et al., 2021). MT1 and MT2 are G–protein coupled receptors associated to Gi/o–proteins, additionally the MT1 receptor can be coupled to a Gq–type protein (Reppart et al., 1996). Activation of the melatonin receptors triggers a set of intracellular signalling responses that lead to an inhibition of the protein kinase A, MT2 is additionally able to inhibit the guanylyl cyclase (GC) (Masana and Dubocovich, 2001; Tosini et al., 2014). Chen and colleagues have stated, that the MT1 receptor can also provide signals through the coupled Gs protein in HEK 293 cells (Chen L. et al., 2014). To this extent, melatonin receptors recruit further downstream messengers, cascades and transcription factors, thereby involving other pathways such as PI3K/PKCζ/c–Raf/MEK/ERK (Chen et al., 2020a), PKC (Soto-Vega et al., 2004), Ca^2+^/CaMKs (Fukunaga et al., 2002; Turjanski et al., 2004), PI3K/Akt (Kong et al., 2008), Sirt1/FOXO1 (Tocharus et al., 2014), SHH (Pan et al., 2021), Hippo (Lo Sardo et al., 2017); for a schematic overview of the pathways see Figure 6; see also Table 1.

## Are the melatonin receptors important for Schwann cell function and neurorepair?

Melatonin deficient pinealectomised rats have had a significantly reduced number of axons and thinner myelin sheaths in sciatic nerves, and restitution of melatonin normalised these parameters (Turgut et al., 2005). Similar phenomena have been observed in pinealectomised chicken—in the sciatic nerve, partial degeneration and vacuolisation of myelin sheaths could be observed (Turgut et al., 2010). The majority of the inbred mouse strains are either melatonin–deficient or secrete the hormone at an insufficient concentration due to the mutated enzyme machinery required for melatonin synthesis, and also the daily concentration of melatonin varies between the strains (Vivien-Roels et al., 1998). For example, the C75Bl6/J strain is melatonin–deficient, (Vivien-Roels et al., 1998), while the A/J strain produces melatonin (Betti et al., 2019). Interestingly, both mouse strains have had differences in the process of Wallerian degeneration (La Hoz et al., 2003). These combined findings suggest that melatonin is important for Schwann cell functioning and neurorepair. Furthermore, luzindole, a selective melatonin receptor antagonist with high affinity to the MT2 receptor, could block activation of the ERK1/2 kinases, which together with melatonin are understood to co–modulate Schwann cell functions (Stazi et al., 2021). Luzindole has impaired regeneration at the neuromuscular junction, but the inhibitor has not affected repair within the sciatic nerve (Stazi et al., 2021), thus implying that melatonin may have distinct functional outcome on regeneration for a particular scenario. Possibly, the melatonin receptors MT1 and MT2 are involved in the modulation of this outcome. Of note, single MT2 and double MT1/MT2 receptor deficient mutant mice have displayed a higher pain sensitivity threshold than controls, whereas knock–out of MT1 had no effect on nociception. However, the MT1–deficient mice have shown deficits in locomotion (Weil et al., 2006). The published data on melatonin receptor deficient strains are scarce and further in–depth ultrastructural studies as well as cell culture–based growth or/and proliferation assays using Schwann cells need to be performed. In this respect, it would be important to discuss whether melatonin affects the expression of growth factors and other morphogens needed for neurorepair.

## Melatonin affects glial cell–derived neurotrophic factor expression in Schwann cells

Interestingly, melatonin upregulates expression of the glial cell–derived neurotrophic factor (GDNF) in Schwann cells to support proliferation and de–differentiation (Tiong et al., 2020). Similarly, melatonin has been demonstrated to upregulate GDNF in neural stem cells (Li et al., 2017), and in the C6 glioma cell line (Armstrong and Niles, 2002). GDNF is responsible for axonal support, survival, remyelination (Chen et al., 2018), and migration of Schwann cells (Heermann et al., 2012) or their precursors (Cornejo et al., 2010); the factor is induced in Schwann cells of injured nerves (Xu et al., 2013). Although many studies have shown that GDNF can promote long–distance regeneration of axons after spinal cord injuries, the uncontrolled expression of GDNF may have side effects owing to an excessive axonal growth, aberrant sprouting, and axonal entrapment resulting in a pathological hypertrophy of the injured nerve (Eggers et al., 2019). For example, overexpression of GDNF induced by lentiviral transduction of Schwann cells has increased their cell density and altered their morphology, which has led to impaired growth of axons and compromised regeneration (Eggers et al., 2019). On the other hand, Eggers and colleagues could methodologically create a precise and controllable GDNF–gradient, which has supported nerve regeneration (Eggers et al., 2019). Based on these combined findings, one could ask whether the melatonin–mediated activation of GDNF occurs in a precisely controlled fashion such that the resulting GDNF gradient would stimulate, rather than hinder, nerve regeneration. Contrariwise, does an overstimulation with melatonin lead to impairment of axonal growth? Overall, the melatonin–mediated GDNF signalling seems to be a mechanism that controls migration of mature or de–differentiating Schwann cells towards the site of lesion, where they enable guided growth and compartmentalisation of the regenerating axons. The melatonin–mediated GDNF signalling, however, may be also supported by other morphogens, such as the Hedgehog proteins, which altogether promote the regenerative process by inducing responses in migrating Schwann cells and growing axonal sprouts. GDNF expression that co–modulates Schwann cell plasticity in regeneration upon melatonin exposition is further supported by the Sonic hedgehog cascade underlying Schwann cell migration.

## Melatonin regulates Schwann cell proliferation and migration *via* the Sonic Hedgehog pathway

Sonic Hedgehog (SHH) is a morphogen mediating signalling pathways involved in mammalian organogenesis, e.g. development of the neural tube (Patten and Placzek, 2000). Aberrant activation of SHH may lead to development of malignant cellular transformations and their migration (Gupta et al., 2010; Riaz et al., 2019; Takabatake et al., 2019). SHH has been considered responsible for proper axonal (re)growth in the spinal cord and in the cortical projections (Harwell et al., 2012), positioning and specification of the neurons in the spinal cord (Yang et al., 2019; Danesin et al., 2021), and for the retinal and cerebellar development. SHH has driven proliferation and migration of different cell types, such as cancer cells (Sari et al., 2018) and oligodendrocyte precursors (Merchán et al., 2007). Furthermore, SHH has been demonstrated to play a significant role in nerve injury: the morphogen has been found upregulated in Schwann cells after nerve injury and has stimulated expression of neurotrophic factors including the brain–derived neurotrophic factor (BDNF) that supports survival and neurite outgrowth in motoneurons (Hashimoto et al., 2008). Additionally SHH, together with MAPK and c–Jun cascades, has been shown to orchestrate differentiation of mature Schwann cells into repair entities in injured nerves (Moreau and Boucher, 2020), see also Table 1 and Figure 6. Pan and colleagues have reported that melatonin influences Schwann cell proliferation and migration *via* the SHH signalling pathway after peripheral nerve injury, as they have observed an increased expression of SHH and the glioma–associated oncogene Gli1 in RSC96 Schwann cells following an *in vitro* melatonin treatment (Pan et al., 2021). Since RSC96 is a transformed cell line, further studies using primary Schwann cells need to be carried out to confirm these results. Nevertheless, SHH has been shown to stimulate in Schwann cells activation of c–Jun, a downstream transcription factor of the MAP kinases (Wagstaff et al., 2020). Therefore, the pathways *via* SHH, c–Jun, and MAPK seem to be interconnected.

## Melatonin regulates Schwann cell proliferation and de–differentiation *via* the Ras/Raf/ERK and MAPK signalling cascade

According to Tiong and colleagues, de–differentiation and proliferation of Schwann cells caused by melatonin rely on the activation of the Ras/Raf/MEK/ERK (MAPK/ERK) pathway (Tiong et al., 2020). Melatonin has been found to upregulate the expression of the MAPK/ERK mediators Ras, p–B–Raf and p–C–Raf, with unchanged expression levels of B–Raf and C–Raf, whereas the p–ERK(p44)/ERK(p44) and p–ERK(p42)/ERK(p42) levels were elevated, and in contrast, the p–SAPK–JNK(p54)/SAPK–JNK(p54), p–SAPK–JNK(p46)/SAPK–JNK(p46) and p–p38/p38 levels were downregulated dose–dependently in the RT4 Schwann cell line (Tiong et al., 2020). SOX2, a transcription factor and a marker for stem cells, was also elevated in RT4 Schwann cells treated with melatonin, indicating that melatonin induces de–differentiation of Schwann cells (Tiong et al., 2020; Govindasamy et al., 2022). Moreover, Chang and colleagues have determined that melatonin simulates proliferation of RSC96 cells by increasing the expression levels of p–ERK1/2 (Chang et al., 2014). The experiments on Schwannoma cells conducted by Tiong et al. and Chang et al. provide first insights into the intracellular melatonin signalling, but it is questionable whether the conclusions can be transferred to non–transformed Schwann cells. Indeed, in perisynaptic Schwann cells at the neuromuscular junction as well as in Schwann cells along the axons of the regenerating sciatic nerve, MT1 has become upregulated, thus leading to an elevated phosphorylation of ERK (Stazi et al., 2021). Although the Schwannoma cells resemble only partially Schwann cells, both cell entities respond to melatonin in a similar fashion.

How does the MAPK/ERK pathway become activated by melatonin? Multiple indirect cascades that do not require Ras have been proposed to explain the activation of the MAPK/ERK pathway by melatonin. Thus, Chen and colleagues have found that in HEK293 cells the melatonin receptors can be coupled to Gs proteins (Chen L. et al., 2014). This coupling leads to activation of the MEK kinase *via* the PKA/PI3K/PDK1/PKC cascade, yet the latter can be counteracted by an inhibitory G–protein which associates with the melatonin receptors (Chen L. et al., 2014). Melatonin has also activated ERK *via* the MT1 or MT2 receptor or the MT1/MT2 heterodimer in HEK293 and NS–1 cells employing the Gβγ/PI3K/PKCζ/c–Raf/MEK/ERK cascade (Chen et al., 2020a). In this respect, Ras, despite its involvement in the melatonin–mediated signalling in Schwann cells, seems to be dispensable for the activation of MAPK/ERK *via* the melatonin receptors (Table 1 and Figure 6).

The Ras/Raf/MEK/ERK signalling pathway has been postulated as a regulator of cell differentiation (Rodríguez-Carballo et al., 2016; Meng et al., 2018), however its sustained activation has induced an opposite effect in Schwann cells, namely de–differentiation (Harrisingh et al., 2004). Cervellini and colleagues have noted that persistent activation of the MAPK/ERK pathway in Schwann cells has stimulated myelination, but in the case of an excessive activation of this pathway de–myelination and de–differentiation of Schwann cells have occurred (Cervellini et al., 2018). Knock–out of ERK1/2 used as a model by Newbern and colleagues has turned out to inhibit myelination of axons, the morphology of the nerves was altered, the ratio of myelinated to non–myelinated axons shifted in favour of the non–myelinated fibres, however no reduction in Schwann cell number occurred, and the myelin basic protein (MBP) and myelin–associated glycoprotein (MAG) were upregulated, whereas the pluripotency related and glial differentiation control proteins Id2 and Id4 were downregulated, all of which has been interpreted by the authors as a premature onset of myelination (Newbern et al., 2011). Surprisingly, this has had little effect on oligodendrocytes, as they have exhibited even more intricate morphology and myelin marker expression than the control littermates (Newbern et al., 2011). Of note, Suo and colleagues have determined that inhibition of the MAPK/ERK pathway by the PD0325901 inhibitor boosts differentiation of neural progenitor cells to oligodendrocytes and promotes myelination, what the authors have proposed as a possible target for therapy of demyelinating diseases such as multiple sclerosis (Suo et al., 2019). Thus, the selective manipulation of the MAPK/ERK pathway may determine the fate of a particular cell population (Lee et al., 2014; Ryu et al., 2015), specifically whether the cells should de–differentiate and proliferate or remain in an arrested cell cycle of terminal differentiation. To sum up, the MAPK/ERK pathway suppresses differentiation, and thus, myelination of Schwann cells, to keep them in a progenitor–like state, capable of high proliferatory activity and being able to differentiate further into distinct subtypes. Nevertheless, the effects of MAPK/ERK depend on the intensity of the activation of this cascade, and different outcomes are possible (Altunkaynak et al., 2018). The role of the MAPK/ERK signalling in the myelination process could explain the findings on improved myelination in injured sciatic nerves *in vivo* (Zhang et al., 2022).

The MAPK/ERK pathway facilitates regulatory influence on gene expression through recruitment of downstream transcription factors—it is known that melatonin can regulate the activity of the c–Jun N–terminal kinases (JNKs) and the transcription factor c–Jun in traumatically injured mouse brains (Rehman et al., 2019), and possibly in breast cancer cells (Chan et al., 2002). The role of c–Jun in myelination, programmed cell death and nerve regeneration is an interesting topic, as it has been described in several studies. The transcription factor c–Jun has been shown to regulate glial phagocytosis of axonal debris through activation of the draper protein in *Drosophila melanogaster* (MacDonald et al., 2013), autophagy and cell death through the regulation of pro– and anti–apoptotic proteins Bcl–2 associated X–protein (Bax), B–cell lymphoma 2 (Bcl–2) (Bogoyevitch et al., 2010). Furthermore, pronounced c–Jun activity has been observed in neuropathies and peripheral nerve lesions (Fazal et al., 2017) and suggested to have both beneficial (Fontana et al., 2012; Hantke et al., 2014; Wagstaff et al., 2020) and maladaptive functions (Parkinson et al., 2008; Huang et al., 2015; Nunes et al., 2021) in regenerating peripheral nerves. As it has been claimed by Blom and colleagues, c–Jun activation in Schwann cells is JNK–independent in the case of peripheral nerve injury and the authors have proposed that other MAPKs are also capable of stimulating c–Jun (Blom et al., 2014). Another study has shown that c–Jun had delayed the onset of myelination (Parkinson et al., 2008) and controlled the phenotypical changes in marker’s expression patterns inside the Schwann cells as they de–differentiate, also controlled neuronal survival, promoted axonal growth and myelin degradation in cooperation with the macrophages during peripheral nerve regeneration (Arthur-Farraj et al., 2012). Activation of c–Jun occurs through N–terminal phosphorylation by ERK1/2 (Leppä et al., 1998; Deng et al., 2012) or predominantly by JNKs at Ser63 and Ser73 (Ruff et al., 2012). c–Jun has been determined to be essential for the regeneration of the mouse facial nerve in deletion experiments performed by Ruff and colleagues, however substitution of the phosphoacceptor sites has shown moderate effects on nerve regeneration, thus suggesting that activation of c–Jun by JNKs might not be the primary mechanism of its regulation during peripheral nerve regeneration (Ruff et al., 2012). Other sites that can be phosphorylated in c–Jun are Ser91, Ser93 and Ser95 (Reddy et al., 2013). Phosphorylation at multiple sites has been determined to initiate pro–apoptotic behaviour of cerebellar granule neurons (Reddy et al., 2013). The Ser95–phosphorylation has been observed to occur after continuous exposure of human embryonic kidney cells to stress, induced by etoposide, which has been proposed as a sensory mechanism of protection against the genotoxicity (Vinciguerra et al., 2008).

## Conclusion

The Schwann cells repair the injured nerve by initiating extracellular matrix reorganisation within the injured region. Each Schwann cell modifies its basal lamina as to accommodate the re–growing axons and, at the same time, clears myelin debris and modulates the composition of the surrounding endoneurium for regrowing axons. In the process of regeneration, the Schwann cells differentiate into an ensemble of various cell types including the myelinating, non–myelinating, phagocytic, repair, and mesenchyme–like phenotypes that provide control upon the regeneration and remyelination process. Since neurorepair requires accurate timing and precision of signalling cascades, the circadian control of Schwann cell functions might represent an essential aspect of neuroregeneration. Indeed, the question of whether an intrinsic rhythm can govern the Schwann cells was raised long time ago. Although the direct experimental evidence for an intrinsic rhythm is missing, the Schwann cells in regenerating nerves respond to alterations in the external clock and react time–dependently to application of melatonin. Melatonin induces remarkable changes in Schwann cells—the hormone promotes de–differentiation, migration, and reduces glial scar formation. These effects seem to influence not only the Schwann cells but also the axo–glial interaction, which is essential for regeneration and remyelination.
